# A Serendipitous Finding of Coagulation Factor VII Deficiency in Two Asymptomatic Patients

**DOI:** 10.7759/cureus.68133

**Published:** 2024-08-29

**Authors:** Nourelhouda Ouerradi, Ghannam Ayad, Aziza Elouali, Abdeladim Babakhouya, Maria Rkain

**Affiliations:** 1 Pediatrics and Child Health, Mohammed VIth University Hospital, Mohammed 1st University Faculty of Medicine and Pharmacy of Oujda, Oujda, MAR; 2 Pediatric Gastroenterology, Mohammed VIth University Hospital, Mohammed 1st University Faculty of Medicine and Pharmacy of Oujda, Oujda, MAR

**Keywords:** pediatric hematology, blood coagulation disorder, blood coagulation factors, asymptomatic, factor-vii deficiency

## Abstract

Congenital Factor VII (FVII) deficiency is a rare autosomal recessive disorder with a prevalence of approximately 1:500,000. It plays a crucial role in initiating coagulation by activating Factors IX and X. Diagnosis typically involves prolonged prothrombin time (PT) and varies widely in clinical presentation. Management includes fresh frozen plasma (FFP), prothrombin complex concentrates (PCC), and recombinant activated FVII (rFVIIa), with rFVIIa often preferred due to its safety and efficacy. We present two pediatric cases: a five-year-old boy with a prolonged PT at 55% and FVII levels at 25.1%, and a two-year-old boy with a PT at 24% and FVII levels at 4.6%. Both cases highlight the importance of thorough hemostatic evaluation and tailored management strategies in FVII deficiency.

## Introduction

Congenital Factor VII (FVII) deficiency is a rare autosomal recessive disorder with a prevalence of approximately 1:500,000. First identified by Alexander et al. in 1951, FVII plays a crucial role in initiating coagulation by activating Factor IX (FIX) and Factor X (FX). The deficiency is categorized into two types: type 1, characterized by reduced functional activity and antigen levels, and type 2, where antigen levels are normal or slightly low, but the protein is dysfunctional [1].

Diagnosis typically involves prolonged prothrombin time (PT). Unlike hemophilia A and B, the correlation between FVII activity and bleeding severity is weak, resulting in a broad clinical spectrum from asymptomatic individuals to those with life-threatening central nervous system hemorrhages. The heterogeneity in clinical presentation is not fully understood.

For managing bleeding episodes, treatment options include fresh frozen plasma (FFP), prothrombin complex concentrate (PCC), activated PCC (aPCC), plasma-derived FVII (pdFVII), and recombinant activated FVII (rFVIIa), with the latter often preferred due to its ease of dosage adjustment and favorable safety profile. Prophylactic treatment, particularly in severe cases manifesting in early childhood, has also been explored with both FFP and rFVIIa [2].

## Case presentation

Case 1

A five-year-old boy from a non-consanguineous marriage was referred from the intensive care unit to the pediatric ward for further evaluation following abnormal hemostasis test results. These tests were performed systematically as part of the preoperative assessment for a planned surgical procedure to correct right testicular ectopy.

The patient's medical history was notable for the absence of bleeding complications post-circumcision or any other bleeding events, and there were no similar cases reported within the family, suggesting no known hereditary bleeding disorders. A comprehensive clinical examination of the patient yielded normal findings.

The initial hemostasis blood tests revealed a normal activated partial thromboplastin time (aPTT) but indicated a prolonged prothrombin time (PT) at 50% of the normal range. Hematocrit levels were within normal limits. These findings suggested a disruption in the extrinsic pathway of hemostasis. Subsequent factor assays confirmed a deficiency in Factor VII, with levels recorded at 25.1% of the normal range (normal values: 70-120%) (Table 1).

**Table 1:** The findings of all the laboratory investigations for case 1

Parameters	Patient values	Normal Range
aPT	50%	70-100%
Hemoglobin	13 g/dL	13-18 g/dL
Hematocrit	37.5%	40-54%
Platelets	395000/µL	150000-400000/µL
Factor VII	25.1%	70-130%

Based on these results, a diagnosis of Factor VII deficiency was established. The clinical presentation and the laboratory findings supported this diagnosis.

To address this coagulation disorder, recombinant activated Factor VII (rFVIIa) was given before and after the planned surgery to correct the right testicular ectopy at 20 µg/kg every 6 hours until hemostasis was secured.

Close monitoring of coagulation parameters and clinical signs of bleeding or complications such as hematomas or excessive surgical site bleeding, was performed regularly, and the patient was discharged after four days.

Case 2

A two-year-and-three-month-old male child presented with abnormal hemostasis test results identified during a pre-anesthetic consultation for an elective circumcision procedure. The hemostasis work-up performed systematically as part of the pre-operative assessment, showed a markedly decreased prothrombin time (PT) at 24%, while the activated partial thromboplastin time (aPTT) was within normal limits, and the complete blood count (CBC) was also normal. These findings suggested a disturbance in the extrinsic pathway of coagulation.

The child was referred to our department for further etiological investigation in response to these abnormal results. A comprehensive somatic examination was performed, which revealed no additional abnormalities, which were otherwise normal.

Further laboratory testing, including specific factor assays, confirmed a severely reduced Factor VII level of 4.6% of the normal range (normal values: 70-120%). This result corroborated the diagnosis of Factor VII deficiency (Table 2).

**Table 2:** The findings of all the laboratory investigations for case 2

Parameters	Patient values	Normal Range
aPT	24%	70-100%
Hemoglobin	13.1 g/dL	13-18 g/dL
Hematocrit	36.1%	40-54%
Platelets	270000/µL	150000-400000/µL
Factor VII	4.6%	70-130%

This patient has not yet undergone the planned elective circumcision. The surgery is scheduled for the upcoming autumn. In preparation for the procedure, a prophylactic treatment plan involving the administration of recombinant activated Factor VII (rFVIIa) has been developed. This regimen will be carefully monitored and adjusted to ensure optimal hemostasis and to minimize surgical risks. Additionally, a trial of vitamin K supplementation will be considered, along with the use of fresh frozen plasma (FFP) as an alternative if necessary. Regular follow-up and comprehensive perioperative care will be integral to the patient's management strategy.

## Discussion

FVII deficiency is the most common among rare coagulation factor deficiencies, with a prevalence of 1:500,000. According to 2008 data from the Turkish Ministry of Health, 240 out of 361 cases of rare coagulation factor deficiencies were FVII deficient; likely due to the high rate of consanguineous marriages [3].

Large series in the literature often include both pediatric and adult patients. The International Registry on Congenital FVII Deficiency (IRF7SG) reported an age range from one to 90 years; this age distribution variability may explain the differences in clinical features [4].

Factor VII (FVII) plays a critical role in the initiation of the coagulation cascade in vivo by activating factors IX (FIX) and X (FX) [5]. The extrinsic pathway of coagulation is initiated at sites of tissue damage through the formation of a complex between activated FVII (FVIIa) and tissue factor (TF), the latter being located in the subendothelium. FVIIa, which constitutes approximately 1% of the total FVII mass, binds to TF, resulting in its activation through cleavage at position 152, forming an FVIIa-TF complex that significantly activates both intrinsic and extrinsic pathways [5]. Congenital FVII deficiency is marked by low levels of circulating FVIIa and presents with a wide range of bleeding symptoms of varying severity, largely due to unknown minimal FVII levels required to prevent bleeding and other uncharacterized factors influencing the clinical picture.

Tissues rich in TF, such as the brain, bowel, uterus, placenta, lungs, and heart, are common bleeding sites in FVII-deficient patients. Unlike hemophilia A or B, where bleeding severity correlates with factor levels, FVII deficiency does not show a clear correlation between circulating FVII levels and clinical manifestations. Bleeding in severe cases can begin within the first six months of life, encompassing life-threatening hemorrhages like intracranial and gastrointestinal bleeding, and recurrent hemarthrosis [6]. Milder cases may present with symptoms triggered by trauma or surgery and can be diagnosed later in life. A notable feature is the higher incidence of symptomatic women, partly due to menorrhagia and minor bleeding events like easy bruising and gum bleeding [7]. Surgical bleeding can be the initial symptom but is less frequent in patients without a history of spontaneous bleeding [8].

Diagnosis of FVII deficiency is typically suspected when a hemostatic screening reveals an isolated prolongation of the prothrombin time (PT) with a normal activated partial thromboplastin time (aPTT). The FVII coagulation activity (FVIIc) assay is crucial for confirming the diagnosis. However, the assay's accuracy can be influenced by the type of thromboplastin used and residual FVII in the substrate, which can affect the sensitivity, particularly at low levels [9]. To enhance the reliability of FVIIc measurements, the use of standardized reference plasma and recombinant thromboplastin has been recommended, reducing inter-laboratory variability [9]. Molecular diagnosis using conventional PCR techniques helps in cases where the clinical picture is unclear or inheritance patterns need clarification. Advanced techniques, such as semi-quantitative multiplex PCR, are employed to detect complex intragenic rearrangements not identified by conventional methods [10].

Management of FVII deficiency involves substitution therapy, especially in severe cases and those with a significant bleeding history. Fresh frozen plasma (FFP), prothrombin complex concentrates (PCC), and recombinant activated FVII (rFVIIa) are commonly used treatments. FFP and PCC have been used historically but carry risks of circulatory overload and thrombotic complications due to the presence of multiple clotting factors [11]. Plasma-derived FVII concentrates, such as PROVERTIN UMTIM3, are effective but have a low risk of blood-borne pathogen transmission due to viral inactivation procedures [11]. rFVIIa (Novoseven) is the most widely accepted treatment, particularly for spontaneous bleeding and surgical settings. It offers a favorable safety profile and efficacy, with an average effective dose of 20 µg/kg administered repeatedly until hemostasis is achieved [11]. However, optimal treatment schedules remain undefined.

Prophylaxis is not commonly practiced in FVII-deficient patients, except in unweaned infants prone to severe and frequent bleedings, and in cases such as menorrhagia with iron deficiency or recurrent hemarthrosis. Continuous infusion of rFVIIa during surgical procedures has also been reported. When properly managed, FVII deficiency generally has a good prognosis, with patients achieving a life expectancy similar to that of the general population. Potential complications of replacement therapy include thrombosis and the development of inhibiting antibodies against FVII, although these events are rare and reported anecdotally [12]. A new formulation of rFVIIa, stable at room temperature (up to 25°C), has been tested in randomized trials, showing equivalence in safety, efficacy, and pharmacokinetics compared to the classic formulation, although assay differences exist for very low plasma levels [12].

Emerging non-replacement therapies, such as gene therapy and pharmacological agents, offer new treatment avenues. Experimental evidence suggests that certain aminoglycosides can suppress premature translation termination caused by nonsense mutations, potentially increasing FVIIc levels. This approach has shown promise in cellular models and in vivo for severe FVII deficiency due to nonsense mutations. The search for more effective molecules to achieve protective levels of the missing factor is ongoing, representing a pioneering challenge in the treatment of congenital clotting defects [13].

## Conclusions

Factor VII deficiency is a rare inherited disorder of hemostasis that can be life-threatening. A minimal hemostasis work-up based on the combination of a low PT and a normal aPTT will point to an abnormality of the exogenous pathway of hemostasis.

Factor VII assay will enable the clinician to distinguish and diagnose factor VII deficiency with certainty. In light of these observations, we would also like to emphasize the fortuitous nature of the disorder, the role of clinician-biologist collaboration for a reasoned biological work-up, and the rarity of this disorder.
